# Huge Pseudoaneurysm of the Femoral Artery after Internal Fixation of Femoral Trochanteric Fracture

**DOI:** 10.1155/2018/3182643

**Published:** 2018-07-25

**Authors:** Hideyuki Kinoshita, Masayuki Hashimoto, Jiro Hirayama, Kouji Fujita, Yoshio Takeuchi, Junichi Iwasaki, Hironori Yamasaki, Mitsuhiro Kitamura, Seiji Ohtori, Tsuguo Morikawa

**Affiliations:** ^1^Department of Orthopaedic Surgery, Graduate School of Medicine, Chiba University, 1-8-1 Inohana, Chuo-ku, Chiba 260-8670, Japan; ^2^Department of Orthopedic Surgery, Chiba Cancer Center, 666-2 Nitonacho, Chuo-ku, Chiba 260-8717, Japan; ^3^Department of Orthopedic Surgery, Chiba Medical Center, 1-7-1 Minamicho, Chuo-ku, Chiba 260-0842, Japan

## Abstract

Pseudoaneurysm is one of the most serious complications of femoral trochanteric fracture surgery. Since the rupture of pseudoaneurysm may lead to death, early detection is important. We report the case of an 80-year-old male who developed pain in the proximal thigh and severe swelling after internal fixation of a femoral trochanteric fracture with a hip nail. Angiography revealed a pseudoaneurysm of a branch of the deep femoral artery near the interlocking screw. The vascular lesion was immediately treated by transcatheter embolization, and the vascular leakage was completely resolved with catheter embolization. After embolization, the patient's clinical state improved rapidly, and the laboratory values improved to normal after several weeks. The current case study reminds us that pseudoaneurysm can occur after intramedullary nail placement to treat a femoral trochanteric fracture.

## 1. Introduction

Femoral trochanteric fracture is a fracture that is often encountered in the clinical setting. One of the most serious complications of femoral trochanteric fracture repair surgery is pseudoaneurysm. Pseudoaneurysm occurring in a branch of the deep femoral artery is a relatively rare complication after surgical repair of a femoral trochanteric fracture. The diagnosis of pseudoaneurysm is usually delayed because of nonspecific clinical features like pain, subcutaneous bleeding, swelling, or unexplained anemia. However, rupture of the pseudoaneurysm may lead to hemorrhagic shock, so a pseudoaneurysm should be suspected early. Moreover, appropriate tests are necessary, such as CT angiography and angiography, for the early detection of pseudoaneurysm. Furthermore, appropriate early treatment intervention such as open surgical repair and coil embolization is necessary to prevent the rupture of the pseudoaneurysm. We present a case of a deep femoral arterial branch pseudoaneurysm diagnosed in an 80-year-old man after the placement of an intramedullary nail for a femoral neck fracture.

## 2. Case Presentation

**Figure 1:**
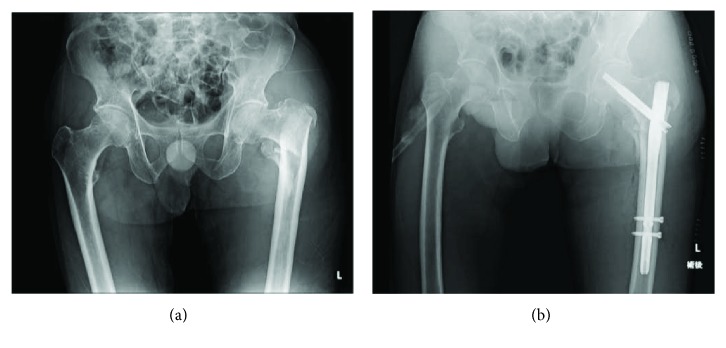
Preoperative (a) and immediate postoperative (b) radiographs show stable intertrochanteric fracture without comminution, acceptable reduction, and good positioning of the nail and screws.

**Figure 2:**
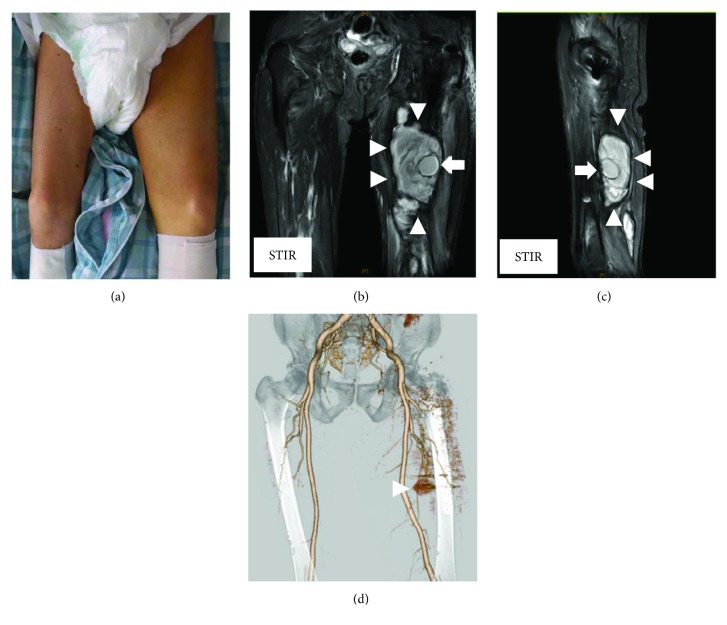
(a) Swelling of the affected limbs. (b, c) Magnetic resonance imaging shows pseudoaneurysms (arrow) and huge hematoma (arrowhead). (d) Computed tomography angiographies with a 3-dimensional reconstruction view demonstrate pseudoaneurysms (arrowhead).

**Figure 3:**
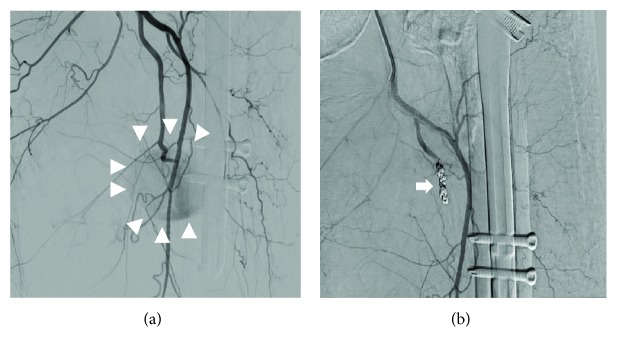
(a) Angiogram demonstrates a pseudoaneurysm of a branch of the profunda femoris artery. (b) Angiogram following endovascular coil embolization (arrow). The angiogram shows no filling of the pseudoaneurysm near the screw.

**Figure 4:**
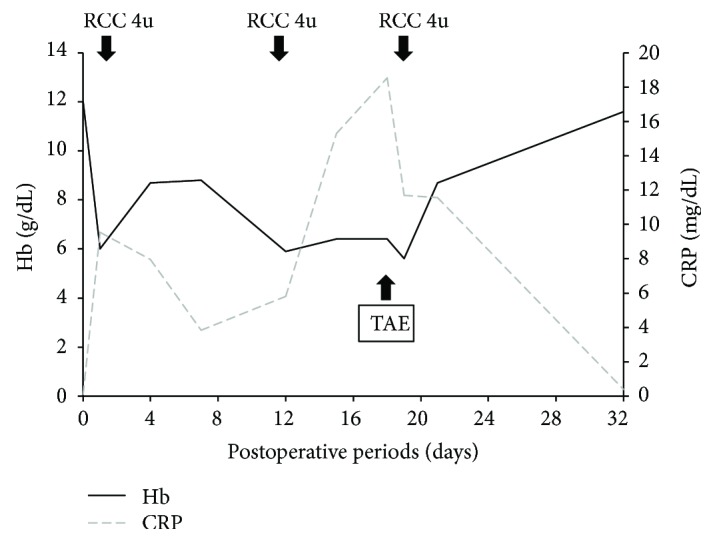
Graph illustrating Hb and CRP levels on consecutive postoperative days.

An 80-year-old man in good health was admitted to our hospital for severe pain in the left hip associated with functional impairment after a fall at home. X-rays and computed tomography (CT) of the painful hip showed a femoral trochanteric fracture with an Evans classification of 1c (Figure 1(a)). Two days after injury, the patient underwent internal fixation with an intramedullary nail via the lateral approach with gentle traction, internal rotation, and adduction using a traction table (Figure 1(b)). The intramedullary nail was fixed with a lag-screw and 2 cortical screws at the distal site (Zimmer® Natural Nail™ System-Cephalomedullary Femoral Nail-Asia short; Zimmer, Warsaw, IN, USA). The operation took 60 minutes, and there was minimal blood loss. The surgical procedure was performed without any intraoperative complications, and there were no changes in his vital signs during the operation. Postoperatively, the patient had no significant clinical problems. However, 1 day after the surgery, his hemoglobin (Hb) value dropped from 12.0 to 6.0 g/dL without any noticeable signs of bleeding, so we transfused 4 units of packed red blood cells to the patient. Four days after the surgery, his Hb value had improved to 8.5 g/dl, but after that, the Hb value continued falling. Twelve days after the surgery, the Hb value dropped to 5.9, and another 4 units of packed red blood cells were transfused. However, the Hb value did not improve. We noticed warmth and subcutaneous bleeding in the left femoral region and suspected an arterial injury due to the surgery (Figure 2(a)). On magnetic resonance imaging (MRI), a huge hematoma was detected in the left inner femoral region (Figures 2(b) and 2(c)). Since pseudoaneurysm due to the surgery was suspected, we performed CT angiography with 3D reconstruction. On CT angiography, a pseudoaneurysm was detected near the tip of the cortical screws at the distal site (Figure 2(d)). After conducting angiography, vascular leakage from a deep femoral artery branch was confirmed (Figure 3(a)). The vascular lesion was immediately treated by transcatheter embolization, and the vascular leakage was completely resolved with catheter embolization (Figure 3(b)). After embolization, the patient's clinical state improved rapidly, and the Hb and C-reactive protein (CRP) values improved to normal after several weeks (Figure 4). One month later, the patient finally left the hospital using a crutch to aid in walking.

## 3. Discussion

Pseudoaneurysms arise from a disruption in arterial wall continuity resulting from inflammation, trauma or iatrogenic causes such as surgical procedures [1]. Pseudoaneurysms in orthopedics may be caused by a fracture, surgery, dislocation, or a wound. In femoral neck fractures, a bone chip at the time of injury can cause the pseudoaneurysm [2]. During surgery, the intramedullary nail used to repair the femur fracture or total hip arthroplasty can cause pseudoaneurysms [3]. Because the deep femoral artery runs along the inside of the femur, it can be easily damaged by hip joint adduction and internal rotation [4]. Therefore, it is necessary to be aware that the deep femoral artery can be damaged by inserting a drill under the hip joint in adduction and internal rotation. Karanikas et al. reported that 3 of 1417 cases of hip fracture surgery could result in pseudoaneurysm [5]. The current case was the first incidence of pseudoaneurysm due to surgery in 300 cases during the past 10 years in our hospital. Age (>60 years), sex (female), diabetes, hypertension, and arteriosclerosis have been reported to be risk factors for pseudoaneurysms [6]. Symptoms of pseudoaneurysm include pain, a pulsatile mass, an audible bruit, a subcutaneous bleeding spot, and increased anemia [7]. The rupture of the pseudoaneurysm may lead to hemorrhagic shock, and early detection and treatment are desirable [8]. Diagnostic testing methods include a supersonic wave, Doppler ultrasonography, CT angiography, MRI, and MRA; furthermore, angiography or percutaneous catheterization is also necessary for testing and treatment. Although the image quality can be impaired by metallic implants, it is a quick and noninvasive method with high sensitivity (90%–95%) and specificity (98%–100%) for detecting arterial injury after trauma [9]. Recently, it was reported that the management of pseudoaneurysms depends mostly on their location and size [10]. Namely, small asymptomatic lesions may be observed for 4–6 weeks with the expectation of spontaneous recovery. On the other hand, in larger (>3 cm) symptomatic lesions, open surgical repair, ultrasound-guided compression, ultrasound-guided thrombin injection, and coil embolization, as seen in this case, are performed.

In the current study, rising CRP levels were also observed with the progression of anemia without signs of infection of the surgical wounds or other organs. CRP levels for early detection are said to correlate with early postoperative complications such as infection, dislocation, and hematoma after proximal femoral fracture surgery [11]. It should be noted that not only infection can develop but also vascular injury, swelling of the affected limb, decreased Hb levels, and elevated CRP levels in the early postoperative period.

In conclusion, we described a case of effective embolization for a pseudoaneurysm of the deep femoral artery caused by an intramedullary nail used to treat a femoral trochanter fracture.
